# Diffusion-based spatial priors for functional magnetic resonance images

**DOI:** 10.1016/j.neuroimage.2008.02.005

**Published:** 2008-06

**Authors:** L.M. Harrison, W. Penny, J. Daunizeau, K.J. Friston

**Affiliations:** Wellcome Trust Centre for Neuroimaging, UCL, London, UK

**Keywords:** Single-subject fMRI, Spatial priors, Weighted graph Laplacian, [non-]stationary spatial process, Diffusion kernel, Eigenmodes, Covariance components, Matrix-variate normal density, Bayesian model comparison, Expectation maximization, Fisher-scoring

## Abstract

We recently outlined a Bayesian scheme for analyzing fMRI data using diffusion-based spatial priors [Harrison, L.M., Penny, W., Ashburner, J., Trujillo-Barreto, N., Friston, K.J., 2007. Diffusion-based spatial priors for imaging. NeuroImage 38, 677–695]. The current paper continues this theme, applying it to a single-subject functional magnetic resonance imaging (fMRI) study of the auditory system. We show that spatial priors on functional activations, based on diffusion, can be formulated in terms of the eigenmodes of a graph Laplacian. This allows one to discard eigenmodes with small eigenvalues, to provide a computationally efficient scheme. Furthermore, this formulation shows that diffusion-based priors are a generalization of conventional Laplacian priors [Penny, W.D., Trujillo-Barreto, N.J., Friston, K.J., 2005. Bayesian fMRI time series analysis with spatial priors. NeuroImage 24, 350–362]. Finally, we show how diffusion-based priors are a special case of Gaussian process models that can be inverted using classical covariance component estimation techniques like restricted maximum likelihood [Patterson, H.D., Thompson, R., 1974. Maximum likelihood estimation of components of variance. Paper presented at: 8th International Biometrics Conference (Constanta, Romania)]. The convention in SPM is to smooth data with a fixed isotropic Gaussian kernel before inverting a mass-univariate statistical model. This entails the strong assumption that data are generated smoothly throughout the brain. However, there is no way to determine if this assumption is supported by the data, because data are smoothed *before* statistical modeling. In contrast, if a spatial prior is used, smoothness is estimated given non-smoothed data. Explicit spatial priors enable formal model comparison of different prior assumptions, e.g., that data are generated from a stationary (i.e., fixed throughout the brain) or non-stationary spatial process. Indeed, for the auditory data we provide strong evidence for a non-stationary process, which concurs with a qualitative comparison of predicted activations at the boundary of functionally selective regions.

## Introduction

Imaging neuroscience now pervades nearly every aspect of neurobiology; from cognitive psychology to neurogenetics. Its principal strength is the ability to make inferences about *structure–function relationships* in the brain. However, statistical parametric mapping (SPM) (Friston et al., 2006), one of the most widely used analyses of brain imaging data, does not support explicit inferences about the spatial aspects of functional anatomy. This is because it uses a mass-univariate approach, which models each voxel (i.e., point in the brain) separately. The need for models that consider influences among voxels, or multivariate models, stems from the fact that neuroimaging data are generated by spatially extended structures that necessarily involve more than one voxel, for example, the organization of retinotopically mapped responses in visual cortex is segregated into distinct cytoarchitectonic areas with defined boundaries. Despite this, it is currently not possible to infer whether a model with non-stationary smoothness (i.e., with boundaries) of functionally selective responses is better than a model with stationary smoothness (i.e., without boundaries).

This paper generalizes and finesses the framework described in Harrison et al. (2007) that allows one to infer the presence of spatially organized responses and evaluate the evidence of different multivariate models of these responses. Critically, we now formulate a spatial prior in terms of the eigensystem of the diffusion kernel of a weighted graph Laplacian (Chung and Yau, 2000). This reduces the computational complexity of the scheme substantially and discloses a clear link with two important methods used to analyze brain imaging data; (i) restricted maximum likelihood (ReML) (Patterson and Thompson, 1974), used to estimate covariance components of a general linear model (GLM) (Friston et al., 2002b) and (ii) Bayesian schemes based on Markov random field (MRF) theory (Bishop, 2006); e.g., the Laplacian priors used in Penny et al. (2005). Furthermore, we generalize the scheme to spatiotemporal models of evoked responses. We demonstrate this by inverting models of functional magnetic resonance imaging (fMRI) time-series data, as opposed to the (second-level) GLMs of static data considered in Harrison et al. (2007). We formulate the problem in terms of diffusion kernels on arbitrary graphs (Grady and Schwartz, 2003) and use them as constraints or empirical priors on the causes (i.e., model parameters) of observed data within a hierarchical Bayesian model. The diffusion kernel can be considered as the covariance of a Gaussian process prior (GPP) (MacKay, 1998). In general, this prior is non-Gaussian as it is embedded on a surface, which encodes local (spatial) geometry of the functional anatomy, i.e., GLM parameter estimates. In this paper, we apply this framework to standard resolution (3 × 3 × 3 mm) fMRI data; however, we expect it to benefit analyses of, for example, high resolution fMRI, diffusion tensor imaging (DTI) and magneto-encephalographic (MEG) data. Indeed, ideas from Harrison et al. (2007) for stationary processes have already been implemented in a model for source reconstruction of MEG data in SPM (Friston et al., 2008).

Critically, this work provides a hypothesis-driven framework; in that a formal model embodies a hypothesis about how we think data are caused. This is important as we develop models that explicitly include spatiotemporal aspects of functional and anatomical principles. These aspects form the basis of *empirical priors* that are optimized in an informed way using the data. In addition, this enables us to formalise the question, “which model do our data support?” using *Bayesian model comparison*. Within a Bayesian paradigm, the intuition is that data are best explained using an optimal balance between model accuracy and complexity. For example, a fine-scaled temporal model of fMRI data is unlikely to enhance temporal feature detection, as its complexity is inappropriate for the coarse sampling rate of fMRI. Bayesian spatiotemporal models allow us to compare models with and without spatially coherent responses and ask whether this coherence is stationary (i.e., the same over space) or not. This sort of inference is central to asking questions about the nature of functional segregation in the cortex, or indeed subcortical structures, such as the amygdala or thalamus.

The potential benefits of this approach are far reaching in that it promises to answer questions, with a measured degree of certainty, about the ‘texture’ and ‘shape’ of functional responses. These questions are becoming increasingly important in imaging neuroscience, for example, investigating midbrain structures such as the periaqueductal gray (Mobbs et al., 2007) in anxiety-related disorders, superior colliculus (Schneider and Kastner, 2005; Sylvester et al., 2007), retinotopic maps of the visual cortex (DeYoe et al., 1994; Engel et al., 1994; Sereno et al., 1994; Warnking et al., 2002) and lateral geniculate nucleus (Haynes et al., 2005), and the fine functional structure within fusiform face area (Grill-Spector et al., 2006). This last example is important as the correspondence that followed this paper indicated that the simple rules used to evaluate the ‘texture’ of response were not correctly formulated, leading to serious criticism of some of their results (Baker et al., 2007; Simmons et al., 2007). A more suitable analysis would be one that models explicitly the spatial features, or geometries, of neuronal responses we want to make inference about.

The paper is organized as follows: the first section motivates the use of multivariate, spatial models in relation to the mass-univariate approach, followed by a brief description of the theoretical fundaments of our approach. We then describe the model in detail with emphasis on using diffusion (heat) kernels to represent covariances within a hierarchical observation model. We provide intuition using synthetic data before applying the approach to fMRI data acquired during auditory processing. We end the paper by discussing some issues with the current implementation and future developments. Details regarding the implementation of the algorithm are given in Appendices A and B.

## Theoretical background

**Fig. 1:**
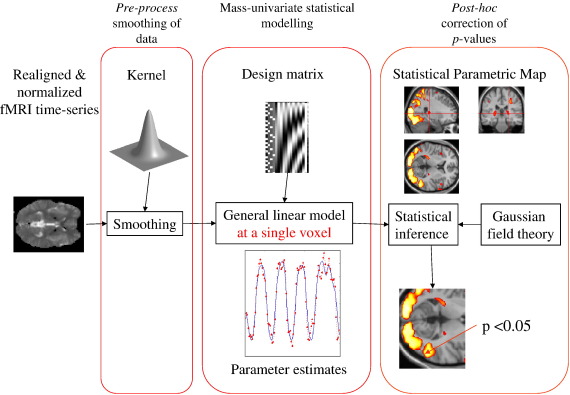
Three-stage procedure in SPM. The statistical model (central panel) models each voxel separately. Several consequences follow; (i) this statistical model is unable to explain correlations in measurements over anatomical space and (ii) inferences over many voxels have to deal with spatial dependencies when adjusting for multiple comparisons. These are dealt with in SPM by smoothing data with a user specified fixed Gaussian kernel (left panel) and using RFT to adjust classical *p*-values *post hoc* (right panel).

To highlight the importance of explicit spatial modeling of neuronal responses, we first consider the mass-univariate approach. A schematic of the data processing stream in SPM (http://www.fil.ion.ucl.ac.uk/spm/) is shown in Fig. 1. This is (excluding the pre-processing steps of realignment, co-registration and normalization) a three-stage procedure. The central panel contains a model of responses at one voxel that can explain data by, and only by, the explanatory variables in the design matrix (upper central panel). As this model is applied to each voxel independently, two extra processing stages are required to accommodate spatial dependencies; smoothing data (left panel) with a user specified kernel and *post hoc* adjustment of *p*-values (right panel), to model spatial dependencies. In this three-stage procedure, spatial properties (that necessarily involve more than one voxel) of neuronal responses are considered before and after modeling *per se*.

Spatially correlated fMRI data cannot be generated from this model, as there are no spatial parameters. As such it is not a generative model of spatially distributed changes in signal. This may seem trivial; however, it entails a deeper issue: in order to test a hypothesis, a data model has to be formulated, which can generate features that are salient to that hypothesis (e.g., temporally structured activity in spatially segregated and functionally selective brain regions). Given this, a prior over GLM parameters (and observation error) can be specified that encodes spatial dependence. The benefit of having an explicit spatial model of GLM parameters is that the three-stage procedure can be subsumed into one generative model. This allows comparison of different spatial models (e.g., stationary vs. non-stationary) and asks which of these has an optimal balance between accuracy (i.e., the expected log likelihood of the model) and complexity (i.e., the number of and uncertainty about parameter estimates). The challenge for requisite *multivariate* models is to embody the general organizational principles of functional segregation and integration (Friston, 2002) into *spatial* models of how data are generated.

This has led to the development of more sophisticated models of fMRI data. Current Bayesian formulations of fMRI spatial models include the stationary Markov random field (MRF) priors of Penny et al. (2005). However, given the convoluted nature of gray matter and patchy functional segregation, a non-stationary model, where the degree of smoothness can depend on spatial location, may be required to model spatial features optimally. A step in this direction has been the use of the multiscale properties of wavelets as a fixed basis set (Flandin and Penny, 2007); however, basis functions that adapt, given local geometric information may provide a more general framework. Non-Bayesian approaches include non-stationary filtering using scale (Siegmund and Worsley, 1995) and rotation spaces (Shafie et al., 2003), Canonical Correlation Analysis (Friman et al., 2003) and edge-preserving bilateral filter kernels; closely related to the diffusion kernel used in this paper, via the Laplace–Beltrami operator ((Polzehl and Spokoiny, 2001; Tabelow et al., 2006; Walker et al., 2006). Although we consider only the simplest noise model in this paper, more realistic models in the literature include non-stationary spatial (Worsley et al., 1999) and stationary spatiotemporal autoregressive models (Penny et al., 2007; Woolrich et al., 2004).

### Spatiotemporal models for fMRI

The framework we propose has its roots in random field theory (RFT) (Adler, 1981; Bishop, 2006), image processing (Geman and Geman, 1984; Sochen et al., 1998; Zhang and Hancock, 2005) and machine learning (MacKay, 1998; Rasmussen and Williams, 2006). As such, we consider *parameter values* of a GLM as a multi-dimensional random field over anatomical space and use graph-based models of diffusion to represent spatial dependence between voxels in a hierarchical Gaussian model. Basically, this entails estimating model parameters of imaging data in the usual way, but coupling the estimation of neighbouring parameters on a graph. This spatial coupling is represented by a spatial prior over nodes (i.e., voxels). Its covariance matrix is given by the diffusion kernel of a graph Laplacian, whose hyperparameters, e.g., dispersion of the kernel, are themselves learnt to provide an anisotropic, non-stationary spatial coherence that is optimized in relation to data.

We use a combinatorial approach to represent a discrete random field instead of discretising a continuous field. The advantage is that we can use standard results from graph theory to formulate the spatial covariance matrix, i.e., the matrix exponential of a graph (or combinatorial) Laplacian (Chung, 1997), which, we think, simplifies the approach and avoids discretising a continuous operator over space. This combination of diffusion on graphs and hierarchical models provides a principled spatial model of the causes of data. It is a natural formulation in terms of kernel methods and probability densities that dissolves the multiple comparisons problem, because there is only one model of the entire image. In this way, we are able to fold pre-process smoothing and *post hoc* correction of *p*-values into the statistical model, i.e., the left and right panels into the central panel in Fig. 1.

### Random fields, Gaussian processes and diffusion

A few words are required in order to explain some of the terminology used above. A ‘random field’ refers to a collection of random variables, typically, over more than one dimension. They can be discrete, e.g., Markov random field, or continuous, e.g., a Gaussian random field, which is specified by a mean and covariance function. This idea can be extended to multi-dimensional random fields, where one or more numbers describe the field at each point in space, e.g., flow. Generalizing further, the field can be on a curved surface, e.g., temperature fluctuations on the two-dimensional surface of an object. This is an example of a continuous random field on a curved manifold. Random fields are exactly the same objects that provide distributional models for the statistics in SPMs and are used to adjust *p*-values in classical mass-univariate analyses of imaging data.

A Gaussian process prior *is* a continuous random field that is used within a Bayesian framework to constrain the estimation of parameters in an observation model e.g., autocorrelation functions over time or GLM parameters over space in a brain volume. GPPs are powerful as they provide (exact) analytic solutions. They are easily generalized to model non-Gaussian processes through specifying a transformation, e.g., log-transform to model a random field of strictly positive numbers (Snelson and Ghahramani, 2007). These have been referred to as ‘warped’ GPPs in the machine learning literature. Generalizing this notion further, a GPP can be defined on any arbitrary surface (sub-manifold), e.g., a cortical surface. We refer to this as an ‘embedded’ GPP.

Diffusion occurs due to the random motion of ‘particles’ within a random field, e.g., molecules in air, and is an example of a local Gaussian process. Diffusion in a continuous media has a discrete analogue on a graph (Chung, 1997) that is comprised of a set of nodes and weighted edges. The Laplacian of a graph is computed using the edge weights, and the diffusion kernel is obtained from the matrix exponential of the Laplacian. This kernel is the solution of the heat equation that propagates a function on nodes of the graph from one moment to another. In other words, the diffusion kernel defines what the function will be at a later time. If the nodes are distributed over space this kernel contains spatial information and can be used as a spatial covariance matrix of a Gaussian density, thereby providing a representation of a discrete random field.

### Hierarchal models and inference

Hierarchical models are at the heart of empirical Bayesian methods used in the analysis of neuroimaging data (Friston et al., 2002a,b). Their appeal is that they provide an intuitive and easily implemented scheme to learn priors, given data. The central idea is that a prior over model parameters can be optimized (or learnt) through further constraints at a higher level. This leads to an observation model comprising levels, or a hierarchy, where each level provides constraints for the one below. Upward and downward passes of sufficient statistics enable learning of priors, given data and as such are called *empirical* priors. Hierarchical models are also used for efficient implementation of model inversion schemes, specifically with large data sets.

RFT is used for topological inference in neuroimaging; i.e., inference about topological features such as at peaks or the Euler characteristic (Worsley et al., 1996). This considers the statistical field, e.g., of classical *t*-values, as deriving from a random field model of the data, where the error terms have a known (or estimable) spatial covariance function. Under this model, null distributions for topological measures (e.g., the Euler characteristic) can be derived and used to adjust associated *p*-values (see Fig. 1). This implicitly controls false positive rates over the search volume. In our Bayesian setting we formulate a model to include a covariance function (matrix for a graph) over both GLM parameters and errors.

The use of RFT, in SPM, can be extended to consider *parameter values* of a generative model as a random field. This acts as a constraint on parameter estimates within a model of data, which itself has to be optimized or learnt; the random field has to be able to change shape for learning to occur, which is enabled by formulating it in terms of a diffusion process. As diffusion processes are locally Gaussian we can treat them as a GPP, which has been used to analyze many diverse types of spatial and temporal (Wang et al., 2005) data, e.g., geostatistics of global weather (Cornford et al., 2005). The appeal is that hierarchies of GPPs can be built within an analytically tractable probabilistic model; a Gaussian process model (Rasmussen and Williams, 2006). In addition they can be used to implement efficient model inversion schemes for large data sets (Quinonero-Candela and Rasmussen, 2005), which make them attractive for modeling neuroimaging data. They can be formulated in terms of graph-theoretic ideas, which provide a discrete representation of a continuous random field on an arbitrary manifold through the weights on a graph. As the graph has a finite number of nodes, this corresponds to a degenerate GPP (Rasmussen and Williams, 2006).

**Fig. 2:**
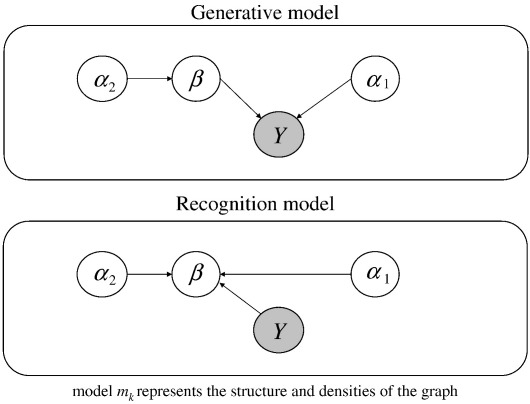
Graphical representation of a generative and recognition model (upper and lower panels respectively). Each node represents a random variable (rv). The observed rv, i.e., data, is shaded and arrows indicate conditional dependence.

A simulated volume of brain data is obtained by sampling from the probability density induced by a hierarchical model. A graphical representation of the generative and implicit recognition models used in this paper is shown in Fig. 2. Nodes and arrows represent random variables and conditional dependence respectively. The model, *m*_*k*_, represents the structure and probability densities of the graph, which is a hypothesis of how data are generated. Parameters of a model, *β*, weight *temporal* explanatory variables are contained in a design matrix. These encode experimental conditions such as auditory stimulus presentation. Each voxel contains a vector resulting in a field of vectors. Hyperparameters, *α*, control the density over these parameters e.g., its *spatial* smoothness. These models can generate synthetic data that contain features similar to those observed in real data. By ‘reversing’ the arrows we can invert the model and use it to *recognize* parameters of the model, given data. This recognition is shown in the lower panel of Fig. 2. The aim is, given data and a model, to estimate the probability density of the causes of data (i.e., model parameters).

This strategy is used to compute the posterior densities over parameters, hyperparameters and the model itself. The latter can be used to compare different models (i.e., hypotheses) of how the data were caused. A simple example of models we would like to compare is stationary vs. non-stationary spatial models. This is important, as it provides a quantitative measure of evidence in favour of one model compared with a competing hypothesis. The posterior over parameters encodes not only the most likely response, over anatomical space, but also a *measure of uncertainty* about the parameters, given data. This probability density can be used to identify patterns of response using posterior probability maps (PPMs) (Friston and Penny, 2003). These are used to visualize structure–function relationships that include a measure of uncertainty after fitting data. Thresholding the posterior density produces a map that represents regions of anatomical space where the probability of parameter values above a threshold has a specified degree of certainty, e.g., regions that have parameter values above zero with probability greater than 0.95. Examples are shown in Figs. 4b and 5e for synthetic and real data respectively. PPMs are important, as they are the basis for inference and hypothesis testing.

## A spatial model for fMRI

In this section, we formulate a two-level GLM in terms of matrix-variate^1^ normal densities (Gupta and Nagar, 2000). In what follows, we will denote a vectorised matrix with an arrow vec(X)=*X*→. Our focus is the formulation of a multivariate normal model, with emphasis on covariance components and their hyperparameters. We start with a linear model, under Gaussian assumptions, of the form

(1) $$\begin{array}{l} Y=X\beta +{ɛ}_{1}\;\;\;\;\;\;\;\;\;\;\;\;\;\;\;\;\;\;\;\;\;\;\;\;\;\;\;\;\;\;\;\;\;\;\;\;\;\;\;p(Y,\beta |X)=p(Y|X,\beta )p(\beta )\\ \beta ={ɛ}_{2}\;\;\;\;\;\;\;\;\;\;\;\;\;\;\;\;\;\;\;\;\;\;\;\;\;\;\;\;\;\;\;\;\;\;\;\;\;\;\;\;\Rightarrow p(Y|X,\beta )=N_{r_{1}}{,}_{c_{1}}(X\beta ,S_{1}⊗K_{1})\\ {ɛ}_{i}∼N_{r_{i}}{,}_{c_{i}}(0,S_{i}⊗K_{i})\;\;\;\;\;\;\;\;\;\;\;\;\;\;\;\;\;\;\;\;\;\;\;\;\;\;\;\;\;\;\;\;p(\beta )=N_{r_{2}}{,}_{c_{2}}(0,S_{2},⊗K_{2}) \end{array}$$

The left-hand expressions specify a hierarchical linear model and the right-hand defines the implicit generative density in terms of a likelihood, *p*(*Y*|*X*, *β*) and prior, *p*(*β*). *N*_*r,c*_ stands for a matrix-variate normal density, where the matrix A ∈ R^r × c^, has probability density function (pdf), *p*(*A*) ~ *N*_*r*__,*c*_(*M*,*S*⊗*K*), with mean, *M*, of size *r *× *c*, and two covariances, *S* and *K*, of size *r *× *r* and *c *× *c*, for rows and columns respectively. Here, Y is a *T *× *N* data matrix and X is a *T *× *P* design matrix with an associated unknown *P *× *N* parameter matrix, *β*, so that *r*_1_ = *T*, *r*_2_ = *P*, *c*_1_ = *c*_2_ = *N*.

The errors at both levels have covariance *S*_*i*_ over rows i.e., time or regressors and *K*_*i*_ over columns i.e., voxels. Eq. (1) is a typical model used in the analysis of fMRI data comprising *T* scans, *N* voxels and *P* parameters. The addition of the second level places empirical shrinkage priors on the parameters. This model can now be simplified by vectorising each component using the identity vec(*ABC*) = (*C*^*T*^ ⊗ *A*)*B*→ (see Appendix A and Harville, 1997).

(2) $$\begin{array}{l} y=Zb+e_{1}\\ b=e_{2}\\ e_{i}∼N_{n_{i}}(0,\Sigma_{i}) \end{array}$$

Where *y* = *Y⃗,* $Z=I_{N}⊗X$, *b* = *β*→⃗, e_i_ = *ε*→⃗_i_, $n_{i}=c_{i}r_{i}$ and $\Sigma_{i}=K_{i}⊗S_{i}$. ⊗ is the Kronecker product of two matrices and **I**_*N*_ is the identity matrix of size *N*. The unknown covariances of the first and second level errors, *Σ*(*α*)_1_ and *Σ*(*α*)_2_, depend on hyperparameters, *α*. The model parameters and hyperparameters are estimated using expectation maximization (EM) by maximizing a lower bound *F*, on the log-marginal likelihood

(3) $$\begin{array}{l} \ln p(y|\alpha )\geq F\\ \;\;\;\;\;\;\;\;\;\;\;\;\;\;\;\;\;\;\;\;\;\;=-\frac{1}{2}(\ln|\Sigma (\alpha )|+y^{T}\Sigma {\left(\alpha \right)}^{-1}y+TN\ln2\pi )\\ \;\;\;\;\;\;\;\;\;\;\;\;\;\Sigma (\alpha )=\Sigma_{1}+Z\Sigma_{2}Z^{T} \end{array}$$

with respect to the parameters, *b*, in the E-step and the covariance hyperparameters, *α*, in the M-step. Here, *Σ*(*α*) represents the covariance of the data induced by both levels of the model. Although the bound in Eq. (3) appears to be only a function of the hyperparameters, we will see later that the form of *Σ*(*α*) can depend on the parameters.

Confounds, such as scanner drift, and mean signal can be conveniently accommodated into the model above by transforming the data. Consider a GLM containing two partitions; one for the signal of interest, *X*_1_, i.e., experimental design matrix, and confounds, *X*_2_, containing a discrete cosine set and column of ones.

(4) $$Y=X_{1}\beta_{1}+X_{2}\beta_{2}+{ɛ}_{1}$$

We can use the change of variables formula (second line, left side of Eq. (5)) to transform this into a more convenient form. Given a function of data, *R*(*Y*), the lower bound is given by

(5) $$\begin{array}{l} \tilde{Y}=R(Y)\\ p(\tilde{Y}|\alpha )=P(Y|\alpha )|J{|}^{\;\;\;\Rightarrow F=-\frac{1}{2}(\ln|\tilde{\Sigma }(\alpha )|+{\tilde{y}}^{T}\tilde{\Sigma }{\left(\alpha \right)}^{-1}\tilde{y}+TN\ln2\pi -2\ln|J|)} \end{array}$$

which now includes an extra term, the Jacobian of the data transformation, *J* = |∂*Y*/*Ỹ*|. Given the transformation, *R*(Y) = *P*_*r*_Y*P*_*c*_, its Jacobian is *J* = |*P*_*r*_|^− *c*^|*P*_*c*_|^− *r*^. If we chose *P*_*r*_ = *I*_*T *_− *X*_2_(*X*_2_*^T^X*_2_)^− 1^ *X*_2_^*T*^, i.e., the projection matrix to the null space of the confounds, and *P*_*c*_ = **I**_*N*_, the model reduces conveniently to one partition, i.e., *Ỹ* = *X̃*_1_*β̃*_1_ + *ε̃*˜_1_, where

(6) $$\begin{array}{l} \tilde{Y}=P_{r}Y\\ {\tilde{X}}_{1}=P_{r}X_{1}\\ {\tilde{\beta }}_{1}=\beta_{1}\\ {\tilde{ɛ}}_{1}∼N_{r_{1},c_{1}}(0,{\tilde{S}}_{1}⊗{\tilde{K}}_{1})\\ {\tilde{S}}_{1}=P_{r}S_{1}P_{r}^{T}\\ {\tilde{K}}_{1}=K_{1} \end{array}$$

In this case, the Jacobian is constant and so we drop the tilde (i.e., by projecting the data and models onto the null space of the confounds, we can proceed as if there were no confounds). However, in general, a data transformation can be parameterized, in which case this term needs to be included in the objective function. The model inversion with EM will be described later (see also Appendix A). First, we look at the hyperparameterization of the spatial covariances and the specific forms of *K*(*α*)_*i*_ entailed by *Σ*_*i*_ = *K*_*i*_ ⊗ *S*_*i*_.

### The spatial priors

In the previous section, we reduced the problem of inverting a linear empirical Bayesian model to optimizing prior covariance components for noise and signal (*i.e*., optimizing the lower bound *F* with respect to the covariance parameters). In this section, we describe diffusion-based priors (Harrison et al., 2007) and consider adaptive priors that are functions of the GLM parameters. In brief, we will assume that the error or noise covariance is spatially unstructured; i.e., *Σ*_1_ = *K*_1_ ⊗ *S*_1_, where *K*(*α*)_1_ = *υ***I**_*N*_ and *S*_1_ = *P_r_P_r_^T^* = *P*_*r*_ (i.e., projection is an idempotent transformation). For simplicity, we will assume that this is fixed over voxels; however, it is easy to specify a component for each voxel, as in conventional mass-univariate analyses.

For the neuronal activity (i.e., signal), we adopt an adaptive prior using a non-stationary diffusion kernel, which is based on a weighted graph Laplacian (Chung, 1997), L(*μ*,*H*), which is a function of the conditional expectation^2^ of parameters, *μ* = 〈*b*〉, and the embedding space metric, *H* (see next section).

(7) $$\begin{array}{l} K{\left(\alpha \right)}_{2}=\exp(-L(\mu ,H)\tau )\\ S{\left(\alpha \right)}_{2}=\eta \end{array}$$

The matrix L is a weighted graph Laplacian, which is a discrete analogue of the Laplace–Beltrami operator used to model diffusion processes on a Riemannian manifold. An example of the latter is the dispersion of heat from a source on the curved surface of a thermally conductive material. Heuristically, this operator propagates quantities locally by dispersing a fixed proportion from each point on a surface or manifold to neighbouring locations. The manifold may itself be embedded in a higher-dimensional space, so that the ensuing diffusion can appear quite complicated. The diffusion kernel is computed using the matrix exponential, which we use as the covariance matrix of a spatial prior. Generally, during optimization the Laplacian is a function of the current image (parameter expectations) and parameters of the embedding space, L(*μ*^(*m*)^,*H*^(*m*)^), where the superscript indicates the *m*th iteration. However, if the Laplacian is approximately constant then *K*_2_^(*m*)^ can be evaluated much more simply (Harrison et al., 2007). This approximation retains the edge-preserving character of the diffusive flow, without incurring the computational cost of reevaluating the Laplacian and its eigensystem. In our experience, weighted graph Laplacians based on the OLS estimate, *μ*_ols_, and an embedding space metric based on its covariance (see Appendix B) give reasonable results.

Hyperparameters of this model comprise, *α* = {*υ*,*τ*,*η*}, where the first hyperparameter controls a stationary independent and identical (i.i.d.) noise component, the second the dispersion of the parameter image and third its amplitude. The row covariance *η* is in general *P *× *P*, where *P* = 1 for all models in this paper. In the next section, we review graph Laplacians and the diffusion model in more detail and then conclude with a summary of the EM scheme used for optimization.

### Diffusion on graphs

Here, we describe diffusion on a graph and illustrate how this is used in a spatial prior. This formulation is useful as it is easily extended to vector and matrix-valued images, which are necessary when modeling a general vector field of parameter estimates, e.g., for a factorial design. We start with some basic graph theory and then discuss diffusion in terms of graph Laplacians. The end point of this treatment is the form of the diffusion kernel, *K*_2_, of the previous section. We will see that this is a function of the parameters that enables the prior smoothness to adapt locally to non-stationary features in the image of parameter estimates.

We consider a graph with vertices (nodes) and edges, *Γ* = (*V*,*E*). The vertex and edge sets are *V* and *E *⊆ *V *× *V*, respectively. An element of each is *v*_*k *_∈ *V* and *e*_*ij *_∈ *E* (note that double indices in subscript distinguish an edge from an error term used in Eq. (2)), where an edge connects two vertices *v*_*i*_ and *v*_*j*_. The total number of nodes and edges are *N_V_* = |*V*| and *N_E_* = |*E*|, where the horizontal bars indicate cardinality, i.e., number of elements in the set. Neighbouring vertices are denoted by *i *~ *j*. Each edge has a weight, *w_ij_*, given by

(8) $$w_{ij}=\{\begin{array}{cc} \exp{(-\text{ds}\left(v_{i},v_{j}\right)}^{2}/\kappa ) & \text{for}\;\;i\sim j\\ 0\;\;\;\;\;\;\;\;\;\;\;\;\;\;\;\;\;\;\;\;\;\;\;\;\;\;\;\;\;\;\;\;\;\;\;\;\;\;\; & \text{otherwise} \end{array}$$

The weights *w*_*ij *_∈ (0,1] encode the relationship between neighbouring voxels and are elements of the weight matrix W, which is symmetric; i.e., *w*_*ij*_ = *w*_*ji*_. They play the role of conductivities, where a large value enables flow between voxels. *κ* is a constant that controls velocity of diffusion, which we set to one. The degree of the *i*th vertex is defined as the sum of all neighbouring edge weights

(9) $$D_{ii}=\sum_{i\sim j}w_{ij}\;\;\;\;\forall \;\;\;\;e_{ij}\;\;\in E$$

The graph Laplacian can be conveniently formulated using results from linear circuit theory (Grady and Schwartz, 2003; Strang, 2004). This has the advantage of representing node and edge spaces explicitly and is defined using the *N*_*E *_× *N*_*V*_ edge-node (see subscript) incidence matrix

(10) $$A_{e_{ij}v_{k}}=\{\begin{array}{c} +1\;\;if\;\;i=k\\ -1\;\;if\;\;j=k \end{array}$$

and *N*_*E *_× *N*_*E*_ constitutive matrix, which is diagonal and contains edge weights, e.g., for the *k*th edge, *C*_*kk*_ = *w*_*ij*_. Given these, the graph Laplacian is

(11) $$L=A^{T}CA$$

This is equivalent to the un-normalized Laplacian of *Γ*, *L* = *D *− *W*, used in Harrison et al. (2007). The weights are a function of the distance, ds(*v*_*i*_,*v*_*j*_), on the surface of a parameter image, *μ*(*u*), between vertices *v*_*i*_ and *v*_*j*_. It is this distance that defines the nature of diffusion generated by the graph Laplacian.

More formally, we specify the distance by choosing a map, *χ*, from the surface of the function *μ*(*u*) to an embedding space, the Euclidean space of $R^{n}$, where *n* = *n*_d _+ *n*_f_ and *n*_d_ and *n*_f_ are the number of spatial and feature dimensions respectively (see Fig. 1; Harrison et al., 2007). Each space has a manifold and metric, (*M*,*g*) and (*N*,*h*), respectively.

(12) $$\begin{array}{l} \chi :M\to N\\ \chi :u\to (\chi^{1}(u),\chi^{2}(u),\chi^{3}(u),\chi^{4}(u))=(u^{1},u^{2},u^{3},\mu (u^{1},u^{2},u^{3})) \end{array}$$

where *n*_d_ = 3, i.e., three spatial dimensions, *n*_f_ = 1, i.e., a scalar field (for the examples in this paper, though this is easily generalized to vector fields) and (*u*^1^,*u*^2^,*u*^3^) are local coordinates. Choosing a metric, *H*, of the embedding space (see below) and computing the Jacobian, *J*, we can calculate the induced metric, *G*, on *μ*(*u*) (Sochen et al., 1998). In matrix form

(13) $$H=\left(\begin{array}{cc} H_{\text{d}} & 0\\ 0 & H_{\text{f}} \end{array}\right)$$

where *H*_d_ is the metric tensor (Frankel, 2004) of the spatial domain. In this paper, we chose this to be Euclidian, i.e., $H_{\text{d}\;}=I_{n_{\text{d}}}$, however, it could be arbitrary, e.g., from a cortical mesh used in anatomically informed models of fMRI or MEG source reconstruction. We fix *H*_f_ to that calculated in Appendix B, based on *μ*_ols_.

The Jacobian (note this term refers to the matrix *and* its determinant) of the map is

(14) $$J=\frac{\partial \chi }{\partial u}=\left(\begin{array}{c} 1\\ 0\\ 0\\ \mu_{u^{1}} \end{array}\begin{array}{c} 0\\ 1\\ 0\\ \mu_{u^{2}} \end{array}\begin{array}{c} 0\\ 0\\ 1\\ \mu_{u^{3}} \end{array}\right)$$

where derivatives are with respect to physical space; i.e., *μ*_*x*_ = ∂*μ* / ∂x, which are computed using central differences. The induced metric, on the surface of *μ*(*u*), is then

(15) $$G=J^{T}HJ$$

which is used to calculate the squared distance

(16) $${\text{ds}}^{2}={\text{du}}^{T}G\text{du}$$

where du = (du^1^,du^2^,du^3^)^*T*^ is displacement in anatomical space. As in general the Laplacian depends on geodesic distance on the embedded sub-manifold of an image we call it a geodesic graph Laplacian (GGL). If $H_{\text{d}\;}=I_{n_{\text{d}}}$ and *H*_f_ = 0 then the Laplacian is based on Euclidean distance in anatomical space. We refer to this as a Euclidean graph Laplacian (EGL). The diffusion kernel can be computed efficiently using the eigenvalue decomposition.

(17) $$\begin{array}{l} L=\Phi \Lambda \Phi^{T}\\ \Lambda =\text{diag}(\lambda_{1},\lambda_{2},\ldots ,\lambda_{N})\\ \Phi =[\phi_{1},\phi_{2},\ldots ,\phi_{N}]\\ K_{2}=\Phi f(\Lambda )\Phi^{T}\\ f(\Lambda )=\exp(-\Lambda \tau ) \end{array}$$

Where the *i*th eigenvalue and vector of the Laplacian are represented by *λ*_*i *_≥ 0 and *ϕ*_*i*_ (a column vector of length *N*) respectively. Given the eigensystem, the matrix exponential can be computed (Moler and Van Loan, 2003) with the added benefit that many other computations are simplified. Related work using the eigensystem of a finite element approximation to the Laplace–Beltrami operator has been used to smooth structural and fMRI data (Qiu et al., 2006) and its diffusion kernel to model cortical thickness and density (Chung et al., 2007). It is instructive to look at the eigenmodes to intuit the covariance components they represent. We will do this by relating them to a restricted maximum likelihood (ReML) (Patterson and Thompson, 1974) based scheme, where the prior covariance, *K*_2_, can be represented using *n* components, *Q*_*i*_ (Friston et al., 2002b).

(18) $$K_{2}=\sum_{i=1}^{n}{\tilde{\lambda }}_{i}Q_{i}$$

The weight of each component, ${\tilde{\lambda }}_{i}$, can then be estimated, given data, using ReML, where there are *n* weights or hyperparameters to estimate. Compare this to an approximation of the diffusion kernel using *n* eigenmodes, where *n* < *N*

(19) $$K_{2}=\sum_{i=1}^{n}\exp(-\lambda_{i}\tau )\phi_{i}\phi_{i}^{T}$$

That is, each eigenmode forms a covariance component, *Q*_*i*_ = *ϕ_i_ϕ_i_^T^*, which is weighted by a function of the Laplacian eigenvalue, i.e.,${\tilde{\lambda }}_{i}=f(\lambda_{i},\tau )=\exp(-\lambda_{i}\tau )$, parameterized by *τ*, which is an eigenvalue of the diffusion kernel. This perspective provides a useful interpretation of the diffusion kernel's eigenspectrum, examples of which are shown in Fig. 4i. Furthermore, it shows that our M-step is formally identical to ReML, when the covariance matrix is given by Eq. (19).

A key difference between the parameterization of the covariance matrices in Eqs. (18) and (19) is that only one hyperparameter, *τ*, has to be estimated in the latter. This is because a functional form (prescribed by diffusion) has been assumed over the weights. This is not the case for Eq. (18) where all *n* weights would have to be estimated separately. This could be achieved easily; however, it does not use information about the spatial process encoded in the spectrum of the Laplacian (i.e., it would not conform to a diffusion prior). An additional benefit of Eq. (19) is that eigenmodes of a GGL represent covariance components that are informed by the (spatial) geometry of GLM parameter estimates (in our case their OLS estimates). We show examples of these eigenmodes (covariance components) for synthetic and real data in Figs. 4f–h and 5h–i.

As seen in Eq. (17) the diffusion kernel is a function of the eigensystem (of the Laplacian matrix). Given a form for the spatial prior that is in terms of a function of the Laplacian eigenspectrum, $p(\beta )=N_{r_{2},c_{2}}(0,S_{2}⊗\Phi f(\Lambda )\Phi^{T})$, the Laplacian prior used in Penny et al. (2005) is recovered using a EGL and *f*(*Λ*) = *Λ*^− 1^, i.e., L is the spatial *precision* matrix, and diffusion-based prior using *f*(*Λ*) = exp(− *Λτ*), where exp(− L*τ*) is a spatial *covariance* matrix. See Appendix A for derivatives, required by the EM scheme, under these priors.

In this paper, we use a reduced eigensystem of *n* = *N* / 10. Note that the spatial covariance matrix afforded by a diffusion kernel is a very large (non-sparse) matrix covering many voxels. This means any reduction helps enormously, in terms of computational load. This reduction produces reasonable results quickly (one slice ~ 2 min using a standard personal computer) and can be motivated gracefully by noting the eigenvalues fall off relatively quickly, due to the fact that diffusion induces smoothness (see Fig. 4i). In the next section, we review briefly the EM algorithm used to optimize the parameters and covariance hyperparameters.

### Expectation maximization

**Fig. 3:**
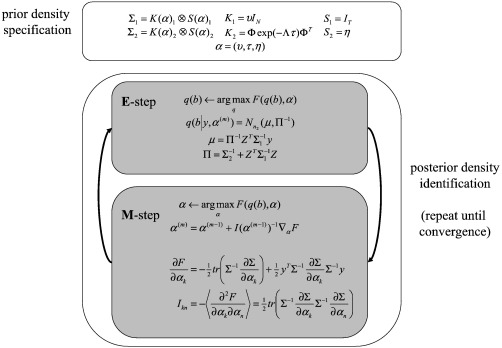
Pseudo-code. Prior densities are specified e.g., diffusion-based prior, and the posterior density optimized, given data, by iterating E and M-steps. The dimension of posterior multivariate density is *n*_2_ = *P* × *N*.

Inversion of the multivariate model in Eq. (2) is straightforward and can be formulated in terms of expectation maximization (EM). EM entails the iterative application of an E-step and M-step (Dempster et al., 1977; Friston et al., 2007; Friston et al., 2002b). Pseudo-code is given in Fig. 3 and expressions for computing all quantities used in the algorithm are provided in Appendix A. We update hyperparameters using a Fisher-scoring scheme.^3^ **I**(*α*) is the expected information matrix, see Wand (2002), with element *I*_*kn*_, where the expectation, 〈 〉, is over the marginal likelihood of the data, *▿_α_F* is the score, i.e., a vector of gradients (where the *k*th element is ∂*F* / ∂*α*_*k*_) with respect to covariance hyperparameters and *Σ* is the current [restricted] maximum likelihood (ReML) estimate of the data covariance.

In summary, to invert our model we simply specify the covariances and their derivatives (see Table 1). These enter an M-step to provide ReML estimates of covariance hyperparameters. *Σ*(*α*)_*i*_ is then used in the E-step to provide the conditional density of the parameters. E and M-steps are iterated until convergence, after which, *F* can be used as a lower bound approximation to the log-evidence or log-likelihood. This represents the accuracy of a model and its complexity, which depend on the number of free parameters and uncertainty in their conditional estimates (see Appendix B; Harrison et al., 2007 and Friston et al., 2007). This means that if two competing models are equally accurate, but one has more free parameters than the other; the model with less parameters has a greater log-evidence. In this way, the procedure embodies the principle of Occam's Razor, “All things being equal, the simplest solution is the best” (MacKay, 2003). This enables comparison of models with a different number of free parameters, as we will see later when comparing models based on different spatial priors. By convention, one requires the difference in log-evidence to be greater then three (i.e., a relative likelihood of about 20:1).

We now have all the components of a generative model that, when inverted, provides parameter estimates that are adaptively smooth, with edge-preserving characteristics. Furthermore, this smoothing is chosen automatically and optimizes the evidence of the model.

## Model comparison

In this section, we compare the performance of three different models of the same data. These models differed in the form of spatial covariance of the prior over voxels; (i) global shrinkage priors (GSP) that are spatially independent, i.e., *K*_2_ = **I**_*N*_; (ii) diffusion kernel of a Euclidean graph Laplacian (EGL) and (iii) diffusion kernel of a geodesic graph Laplacian (GGL). Each model was optimized given synthetic and real fMRI data using the EM algorithm described above. Parameter estimates, posterior probability maps^4^ (PPMs) and model evidences were compared as described next.

### Synthetic data

**Fig. 4:**
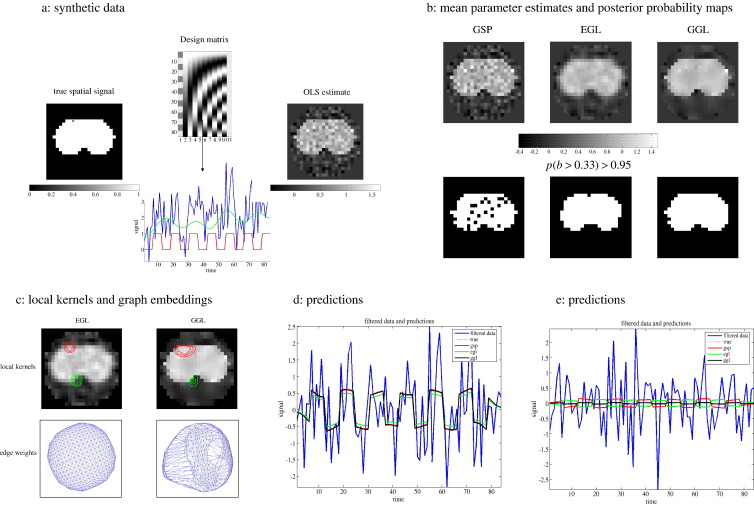

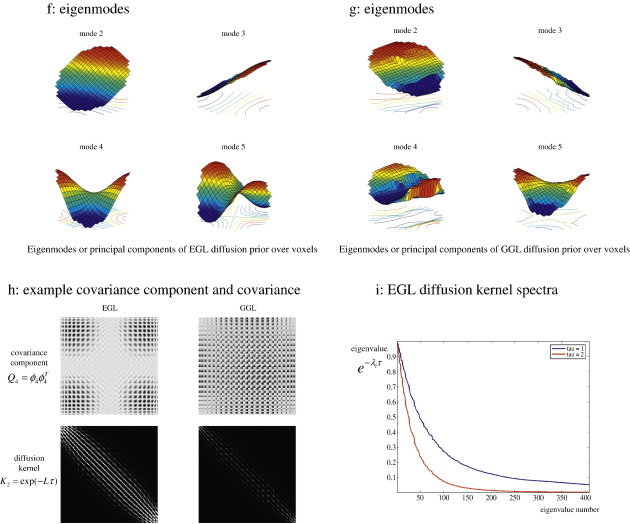
Synthetic data. Data were simulated using a generative model with non-stationary spatial kernel, producing two distinct regions. (a) design matrix (top), true spatial signal (left), example time-series (lower) and OLS estimate of first column GLM parameters (right), (b) posterior mean estimates of GSP, EGL and GGL-based priors on top row and PPMs, threshold at *p*(*b* > 0.33) > 0.95, below, (c) local kernels of EGL and GGL on top row along with plot of edge weights below, (d, e) predictions against data at two locations (same as local kernels in panel, c) inside [outside] the edge of the central region, (f, g) 2nd–5th eigenmodes of EGL and GGL (1st eigenmode is not included as it is constant over the graph), (h) outer product of 4th eigenmode (covariance component) on top row and full diffusion kernel (sum of all covariance components weighted by their eigenvalues) below, and (i) spectra of EGL at two values of *τ*.

Synthetic data are shown in Fig. 4, where a known two-dimensional spatial signal (shown on the left of Fig. 4a) and design matrix containing temporal components (top of 4a), were used to simulate data. The design matrix contains two partitions; the first column is the effect of interest, which is weighted by the known spatial signal, while the remaining columns represent confounds and contain low frequency oscillations used to simulate scanner drift and a mean term. An example of an observed time-series (blue) from the marked pixel is shown below. This comprised a component of interest (red), confounds (green) and i.i.d. Gaussian noise. The ordinary least squares (OLS) estimates of the signal of interest, i.e., image of parameter estimates for the first column, are shown on the right of Fig. 4a. Compare this with posterior mean estimates from GSP, EGL and GGL-based spatial priors shown in Fig. 4b, along with PPMs, thresholds at *p*(*b* > 0.33) > 0.95. The differences are clear, with poor recovery using GSP, blurred mean with rounded edges of the central image with EGL and preservation of the majority of this edge using GGL. Two kernels,^5^ which encode spatial dependences between a pixel (marked with an open circle) and others in its neighbourhood, are shown in Fig. 4c along with a plot of edge weights that uses the second and third eigenvectors as coordinates. Each line segment of this plot represents an edge of the graph and is a useful way to view the [an]-isotropy of a Laplacian matrix. These reveal spatial features of the OLS parameter estimates encoded in the GGL that are not present for EGL. Predictions from the two marked pixels in Fig. 4c are shown in Figs. 4d and e. Fig. 4e demonstrates detection of spurious signal that is not present in the data using GSP and EGL. This does not occur using GGL.

Eigenmodes of EGL and GGL are shown in Figs. 4f and g respectively (formatted as images). Note that the first eigenmode is not shown as this is constant over the graph. These can be regarded as components of the empirical prior covariance over voxels. They provide insight into the feature preserving nature of GGL; note the central region of the OLS parameter image is encoded in its eigenmodes, which means that parameters at two locations (a fixed distance apart) within the central region are more likely to covary, compared to when one location is outside this region. As such, they are non-stationary functions over the graph. This is not the case for EGL, whose eigenmodes are stationary. An example covariance component (fourth eigenmode) is shown in the top row of 4h and full diffusion kernel (i.e. the sum of all eigenmodes weighted by their eigenvalues) below. Spectra, i.e. eigenvalues, $e^{-\lambda_{i}\tau }$, of the EGL diffusion kernel are shown in Fig. 4i for two different values of *τ*. This shows dependence of the spectrum on *τ*. Note the rapid decay with larger *τ*. Eigenmodes with small eigenvalues contribute little to the total covariance matrix; this is the rationale for using a reduced eigensystem. The results of Bayesian model comparison are given in Table 2 and confirm that the evidence for the GGL-based prior is largest, which concurs with the known non-stationarity of the data set.

### fMRI data

**Fig. 5:**
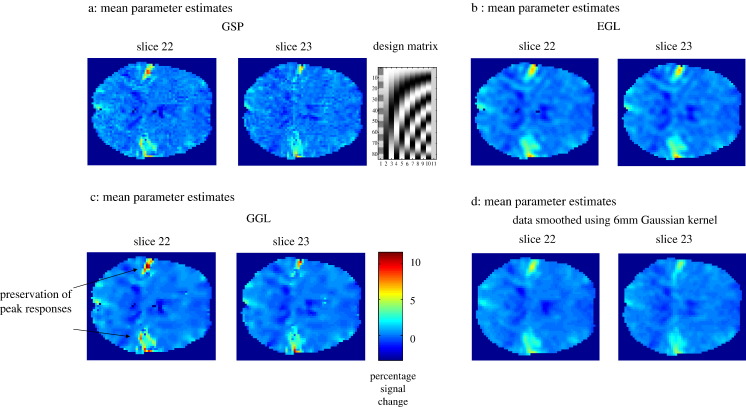

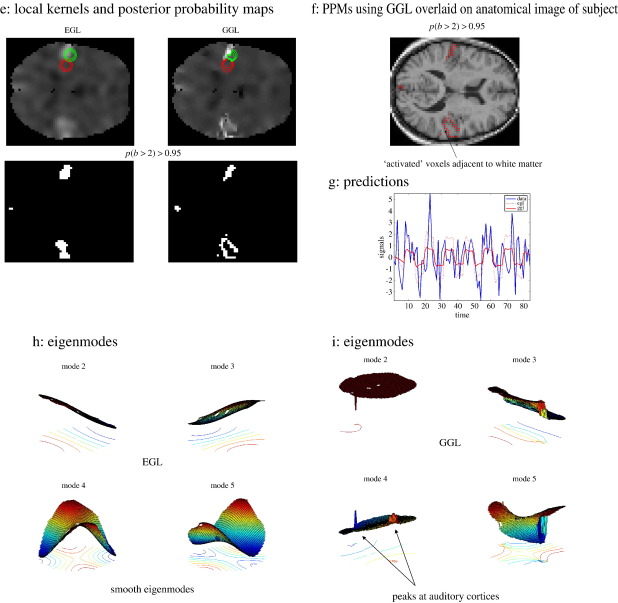
Real fMRI data. Mean parameter estimates (two slices through auditory cortex) of standard resolution fMRI (3 mm^3^) data of one subject's response to an auditory stimulus. (a–c) Posterior mean estimates using GSP, EGL and GGL respectively, (d) data smoothed with a 6 mm Gaussian kernel (conventional practice), (e) local kernels and PPMs, threshold at *p*(*b* > 2) > 0.95, (f) PPM for GGL overlaid on anatomical image (same resolution as functional data), (g) comparison of predictions from EGL and GGL models and (h, i) 2nd–5th eigenmodes (in image format) from EGL and GGL.

Results for fMRI data collected during auditory stimulation are shown in Fig. 5. These data are available freely at http://www.fil.ion.ucl.ac.uk/spm/data/auditory.html and were pre-processed as described in the SPM manual, with the exception of not smoothing data. A simple design matrix with two partitions (auditory stimulus and confounds) was used (see design matrix in upper right of Fig. 5a). This is a very simple experimental design, with the effect of interest encoded in the first column. This means that parameter estimates of this effect form a scalar field over anatomical space. The main effects of auditory input (first column), from two slices (22 and 23 of 46) through the auditory cortex, are shown in Figs. 5a–c for GSP, EGL and GGL-based spatial priors respectively. For comparison, we include mass-univariate parameter estimates in Fig. 5d, using the conventional practice of smoothing data with a 6 mm Gaussian kernel. Compared with conventional smoothing of the data, differences in estimated responses in Figs. 5a–c are clear, with noisy estimates in Fig. 5a, smooth parameter images in Fig. 5b and less attenuation of signal at peaks in Fig. 5c, along with smooth estimates within quiescent regions (the colour scale beneath the images indicates percent signal change). This is due to the border-preserving nature of the non-stationary prior, which allows the degree of smoothness of a parameter image to vary over space. This means that parameter images look sharper, as edges between functionally segregated regions are preserved and not blurred by the constraint of stationarity.

Bayesian model comparison revealed the non-stationary GGL model in Fig. 5c had the greatest evidence (see Table 2). This model was able to extract the structured deployment of cortical responses that are otherwise blurred by EGL. Note that this comparison could not have been made if data were smoothed outside the statistical model. Local kernels and PPMs, i.e., maps of voxels where the model is 95% sure that the effect size is greater than 2% of the global mean (for slice 22 of 46), are shown for EGL and GGL in Fig. 5e. PPMs represent statistical inferences with clear differences in that ‘active’ voxels using EGL are reduced to ‘blobs’, whereas filamentous responses are recovered for GGL, corresponding to their genesis in gray matter. This difference is crucial as decisions regarding data are based on such inferences. The PPM using GGL is shown in Fig. 5f overlaid on an anatomical image (at the same resolution as functional data). White matter has, in general, a lighter shade in this image, which shows ‘activations’ adjacent to white matter and concurs qualitatively with our expectation that BOLD signal has a cortical origin.

Predictions from EGL and GGL-based models are shown in Fig. 5g at the boundary of response in the left auditory cortex (at the location of the green kernel in Fig. 5e). These show a poor fit for EGL suggesting that the isotropy assumption is inappropriate for these data. Eigenmodes (in image format) from EGL and GGL in Figs. 5h–i show peaks in the auditory regions for GGL, but not for EGL. Again, these reveal the non-stationary nature of the GGL-based spatial covariance, compared with EGL.

## Discussion

We have outlined a Bayesian scheme to estimate the optimal smoothing of conditional parameter estimates, given a diffusion-based spatial prior and have applied it to single-subject fMRI data. The contrast between stationary and non-stationary spatial models is remarkable and suggests that the isotropic assumption implicit in conventional smoothing is not appropriate for these data. We have shown this formally using Bayesian model comparison and qualitatively by comparing predictions at a functional boundary. Our approach provides a principled way to compare assumptions about the spatial nature of data that would otherwise not be possible using the standard approach of smoothing data at a pre-processing stage of analysis. Diffusion-based spatial priors allow the strong assumption of isotropy to be relaxed. This is important as the brain is comprised of functional structures that have different spatial scales e.g., cortical and subcortical.

Formulating the model in terms of the eigenmodes of a weighted graph Laplacian allows us to make contact with classical covariance component estimation, i.e., ReML-based schemes (Friston et al., 2002b; Patterson and Thompson, 1974) and conventional Laplacian priors (Penny et al., 2005). Given these eigenmodes, the emphasis is then on finding a parameterized function of their eigenvalues that best explains data; for example, the diffusion-based prior in this paper uses a function parameterized by *τ*, i.e., *f*(*Λ*,*τ*) = exp(− *Λτ*). This diffusion kernel specifies a spatial process where the shape of a local neighbourhood is represented by edge weights and whose scale is controlled by *τ*. This reduces the problem to optimizing *τ*, which also produces compelling results of the sort reported above.

The usefulness of the Laplacian eigensystem has also been explored in regularization schemes for image restoration and smoothing. However, there is a substantial distinction between regularization and Bayesian modeling. Regularization parameters control the effective complexity of a model and determine the degree of over-fitting (Bishop, 2006), whereas Bayesian schemes provide a principled approach to represent and estimate uncertainty of such parameters, using hierarchical models. As such the Bayesian paradigm provides a powerful framework, where model complexity is included in an estimate of the probability of data, given the model, e.g., where models may differ depending on the form of prior used to embody a hypothesis about how data are generated.

In our scheme, data are not regularized (smoothed). Instead model parameters are represented as random fields that have, in general, non-stationary smoothness. The aim is not only to estimate a posterior density on these fields, but also to estimate optimal regularization parameters, such as the dispersion of a diffusion kernel. This enables comparison of different hypotheses about the data; e.g., what are the odds that a non-stationary spatial process generated the data compared with a stationary process. Given this, we consider the material in this paper to go beyond simple regularization schemes based on the Laplace–Beltrami operator.

We have reported only two slices of data analyzed using our approach, which reflects an outstanding issue. As there is only one model of the data, there is just one Laplacian, which is over all voxels in the brain. The associated spatial prior corresponds to a covariance matrix of the order 10^5^, which is computationally prohibitive for current standard personal computers. This is a general implementation issue for Gaussian process priors that require inversion of large matrices. The current implementation of Penny et al.'s algorithm in SPM processes one slice at a time, meaning that a 2D Laplacian is used instead of 3D. While data are measured slice by slice, the underlying functional anatomy is in general 3D, which suggests that 3D models are appropriate. A possible solution is to use a weighted graph Laplacian to partition (Grady and Schwartz, 2003; Qui and Hancock, 2005) a brain volume into computationally manageable pieces. A diffusion-based prior would then be used for each partition independently. Another approach, which we are currently exploring, is to generate data on, and only on, the cortical surface. This generative model could be used to explain observed responses that have been assigned to the cortical mesh using anatomically informed basis functions (Kiebel et al., 2000). Alternatively, the model could generate 3D data by diffusing the 2D cortical response over a 3D mesh. This would have the advantage of conforming to the known anatomical generation of BOLD signal, requiring smaller prior covariance matrices, while modeling full 3D image data.

## Figures and Tables

**Table 3:** Column precisions ${\overset{―}{K}}_{a1b}^{-1}=\Phi_{K_{a}}^{T}K_{1}^{-1}\Phi_{K_{b}}$, where *a*,*b *⊂ {1,2}

	1	2
1	$\Phi_{K_{1}}^{T}K_{1}^{-1}\Phi_{K_{1}}$	$\Phi_{K_{1}}^{T}K_{1}^{-1}\Phi_{K_{2}}$
2	$\Phi_{K_{2}}^{T}K_{1}^{-1}\Phi_{K_{1}}$	$\Phi_{K_{2}}^{T}K_{1}^{-1}\Phi_{K_{2}}$

**Table 4:** Row precisions $\overset{―}{S}$_a1b_^–1^, where *a*,*b *⊂ {1,2}

	1	2
1	$\Phi_{S_{1}}^{T}S_{1}^{-1}\Phi_{S_{1}}$	$\Phi_{S_{1}}^{T}S_{1}^{-1}X\Phi_{S_{2}}$
2	$\Phi_{S_{2}}^{T}X^{T}S_{1}^{-1}\Phi_{S_{1}}$	$\Phi_{S_{2}}^{T}X^{T}S_{1}^{-1}X\Phi_{S_{2}}$

**Table 5:** Eigenvalues of derivatives (with respect to γ = ln α)

	1	2	3
*dD_a_*^(*k*)^	$D_{K_{1}}⊗D_{S_{1}}^{}$	$-\Lambda_{2}D_{K_{2}}\tau ⊗D_{S_{2}}$	$D_{K_{2}}^{}⊗D_{S_{2}}^{}$

**Table 1:** Derivatives of data covariance matrix (using *γ* = ln*α*)

*K*	1	2	3
Hyperparameter	*υ*	*τ*	*η*
$\frac{\partial \Sigma }{\partial \gamma_{k}}$	$\frac{\partial K_{1}}{\partial \gamma_{1}}⊗S_{1}$	$\frac{\partial K_{2}}{\partial \gamma_{2}}⊗XS_{2}X^{T}$	$K_{2}⊗X\frac{\partial S_{2}}{\partial \gamma_{3}}X^{T}$
${\tilde{A}}_{a}^{\left(k\right)}$	*K*_1_	− L*K*_2_*τ*	*K*_2_
${\tilde{B}}_{a}^{\left(k\right)}$	*S*_1_	*XS*_2_*X*^*T*^	*XS*_2_*X*^*T*^

**Table 2:** Model comparison for synthetic (Fig. 4) and real data (Fig. 5)

Covariance	Synthetic data	Real data
GSP	− 46,891	− 36,891
EGL	− 46,629	− 36,292
GGL	− 46,488^⁎^	− 35,150^⁎^

Log-evidence for GSP, EGL and GGL. Greatest evidence indicated by ^⁎^.

## Appendix A Linear algebra for the EM scheme

This appendix provides notes on the linear algebra used to compute the gradients and curvatures necessary for the EM scheme in the main text. They are not necessary to understand the results presented above but help optimize implementation.

We require the bound on the log-marginal likelihood, ln *p*(*y*|*α*) and its derivatives.

(A.1) $$\begin{array}{c} F=-\frac{1}{2}\left(\ln\left|\Sigma \right|+y^{T}\Sigma^{-1}y\right)+\text{const}\\ \Sigma (\alpha )=\Sigma_{1}+Z\Sigma_{2}Z^{T}\\ \Sigma_{i}=K_{i}⊗S_{i} \end{array}\text{.}$$

The first term of Eq. (A.1) is

(A.2) $$\begin{array}{c} \ln\left|\Sigma \right|=\ln\left|\Sigma_{1}\right|+\ln\left|\Sigma_{2}\right|+\ln\left|\Sigma_{2}^{-1}+Z^{T}\Sigma_{1}^{-1}Z\right|\\ =\ln\left|\Sigma_{1}\right|+\ln\left|\Sigma_{2}\right|+\ln\left|\Pi \right| \end{array}$$

where $\left|Z+UWV^{T}\right|=\left|Z\right|\left|W\right|\left|W^{-1}+V^{T}Z^{-1}U\right|$, see appendix of Rasmussen and Williams (2006). This can be reduced further using $\left|\Sigma_{i}\right|=\left|K_{i}⊗S_{i}\right|={\left|K_{i}\right|}^{\text{rank}(S_{i})}{\left|S_{i}\right|}^{\text{rank}(K_{i})}$. The second term is

(A.3) $$\begin{array}{c} y^{T}\Sigma^{-1}y=\text{tr}(Y^{T}A_{{ɛ}_{1}})\\ A_{{ɛ}_{1}}=S_{1}^{-1}{\hat{ɛ}}_{1}K_{1}^{-1} \end{array}$$

where we have used vec(A)^T^vec(B) = tr(A^*T*^B) and, where εˆ_1_ = Y-Xβˆ is the matrix of prediction errors, where μ = vec(βˆ).

### A.1 Conditional moments of parameters (E-step)

The conditional precision is

(A.4) $$\Pi =Z^{T}\Sigma_{1}^{-1}Z+\Sigma_{2}^{-1}=K_{1}^{-1}⊗X^{T}S_{1}^{-1}X+K_{2}^{-1}⊗S_{2}^{-1}\text{.}$$

The conditional covariance can be formulated in terms of eigenmodes of the second level prior covariance as follows: using the matrix inversion lemma, ${\left(Z+UWV^{T}\right)}^{-1}=Z_{}^{-1}-Z_{}^{-1}U{\left(W_{}^{-1}+V^{T}Z_{}^{-1}U\right)}^{-1}V^{T}Z_{}^{-1}$, the data precision is

(A.5) $$\begin{array}{c} \Sigma^{-1}=\Sigma_{1}^{-1}-\Sigma_{1}^{-1}Z{\left(\Sigma_{2}^{-1}+Z^{T}\Sigma_{1}^{-1}Z\right)}^{-1}Z^{T}\Sigma_{1}^{-1}\\ =\Sigma_{1}^{-1}-\Sigma_{1}^{-1}Z\Pi^{-1}Z^{T}\Sigma_{1}^{-1} \end{array}\text{.}$$

Using the eigenvalue decomposition; Σ_2_ = Φ_2_*D*_2_Φ_2_^*T*^, where $\Phi_{2}=\Phi_{K_{2}}⊗\Phi_{S_{2}}$, $D_{2}=D_{K_{2}}⊗D_{S_{2}}$, i.e., *Φ* and *D* are eigenvectors and eigenvalues respectively, then

(A.6) $$\begin{array}{c} \Sigma =Z\Phi_{2}D_{2}\Phi_{2}^{T}Z^{T}+\Sigma_{1}\\ \Sigma^{-1}=\Sigma_{1}^{-1}-\Sigma_{1}^{-1}Z\Phi_{2}{\left(D_{2}^{-1}+\Phi_{2}^{T}Z^{T}\Sigma_{1}^{-1}Z\Phi_{2}\right)}^{-1}\Phi_{2}^{T}Z^{T}\Sigma_{1}^{-1} \end{array}\text{.}$$

Comparing the last line of Eq. (A.5) with (A.6)

(A.7) $$\begin{array}{c} \Pi^{-1}=\Phi_{2}E\Phi_{2}^{T}\\ E={\left(D_{2}^{-1}+\Phi_{2}^{T}Z^{T}\Sigma_{1}^{-1}Z\Phi_{2}\right)}^{-1}\\ =D_{2}^{1/2}{\left(I+D_{2}^{1/2}\Phi_{2}^{T}Z^{T}\Sigma_{1}^{-1}Z\Phi_{2}D_{2}^{1/2}\right)}^{-1}D_{2}^{1/2} \end{array}\text{.}$$

Note, for a diffusion-based prior, $D_{K_{2}}=f(\Lambda )=\exp(-\Lambda \tau )$, however, we could use $D_{K_{2}}=f(\Lambda )=\Lambda_{}^{-1}$ for a Laplacian prior (numerically stable expressions for each are given in the last and penultimate lines of Eq. (A.7) respectively).

The conditional mean is

(A.8) $$\mu =\Phi_{2}E\Phi_{2}^{T}Z^{T}\Sigma_{1}^{-1}y$$

### A.2 Conditional moments of hyperparameters (M-step)

To compute the derivatives^6^ required for the M-step, we use standard results for Kronecker tensor products to show the score and expected information reduce to

(A.9) $$\frac{\partial F}{\partial \gamma_{k}}=-\frac{1}{2}tr(A_{a}^{\left(k\right)}⊗B_{a}^{\left(k\right)}-(F_{a}^{\left(k\right)}C⊗G_{a}^{\left(k\right)}D)E+A_{ɛ}^{T}{\tilde{B}}_{a}^{\left(k\right)}A_{ɛ}{\tilde{A}}_{a}^{{\left(k\right)}^{T}})$$

and

(A.10) $$\begin{array}{c} \frac{\partial^{2}F}{\partial \gamma_{k}\partial \gamma_{n}}=\frac{1}{2}\text{tr}(A_{a}^{\left(k\right)}A_{b}^{\left(n\right)})\text{tr}(B_{a}^{\left(k\right)}B_{b}^{\left(n\right)})+\\ \frac{1}{2}\text{tr}\left((F_{b}^{\left(n\right)}C⊗G_{b}^{\left(n\right)}D)E(F_{a}^{\left(k\right)}C⊗G_{a}^{\left(k\right)}D)E\right)-\\ \frac{1}{2}\text{tr}\left((F_{b}^{\left(n\right)}A_{a}^{\left(k\right)}C⊗G_{b}^{\left(n\right)}B_{a}^{\left(k\right)}D)E\right)-\frac{1}{2}\text{tr}\left((F_{a}^{\left(k\right)}A_{b}^{\left(n\right)}C⊗G_{a}^{\left(k\right)}B_{b}^{\left(n\right)}D)E\right) \end{array}$$

where the superscript of matrices *A*, *B*, *F*, and *G* represents a hyperparameter index, i.e., *k* ⊂ {1,2,3}, while the subscript represents a level index for error covariances, i.e., *a* ⊂ {1,2}, which will simplify expressions later. Terms in Eqs. (A.9) and (A.10) are given by

(A.11) $$\begin{array}{c} \frac{\partial F}{\partial \gamma_{k}}=\frac{\partial \Sigma }{\partial \lambda_{k}}\\ A_{a}^{\left(k\right)}=K_{1}^{-1}{\tilde{A}}_{a}^{\left(k\right)}\\ B_{a}^{\left(k\right)}=S_{1}^{-1}{\tilde{B}}_{a}^{\left(k\right)}\\ C=K_{1}^{-1}\Phi_{K_{2}}\\ D=S_{1}^{-1}X\Phi_{S_{2}}\\ E=D_{2}^{1/2}{\left(I+D_{2}^{1/2}\Phi_{2}^{T}Z^{T}\Sigma_{1}^{-1}Z\Phi_{2}D_{2}^{1/2}\right)}^{-1}D_{2}^{1/2}\\ F_{a}^{\left(k\right)}=\Phi_{K_{2}}^{T}K_{1}^{-1}{\tilde{A}}_{a}^{\left(k\right)}\\ G_{a}^{\left(k\right)}=\Phi_{S_{2}}^{T}X^{T}S_{1}^{-1}{\tilde{B}}_{a}^{\left(k\right)} \end{array}\text{.}$$

Supporting calculations for the score are

(A.12) $$\begin{array}{c} \frac{\partial F}{\partial \gamma_{k}}=-\frac{1}{2}\left(\text{tr}(\Sigma^{-1}\frac{\partial \Sigma }{\partial \lambda_{k}})+y^{T}\Sigma^{-1}\frac{\partial \Sigma }{\partial \lambda_{k}}\Sigma^{-1}y\right)\\ \frac{\partial F}{\partial \gamma_{k}}=\text{tr}\left((\Sigma_{1}^{-1}-\Sigma_{1}^{-1}Z\Pi^{-1}Z^{T}\Sigma_{1}^{-1}){\tilde{A}}_{a}^{\left(k\right)}⊗{\tilde{B}}_{a}^{\left(k\right)}\right)\\ =\text{tr}\left(A_{a}^{\left(k\right)}⊗B_{a}^{\left(k\right)}-(C⊗D)E(F_{a}^{\left(k\right)}⊗G_{a}^{\left(k\right)})\right)\\ =\text{tr}\left(A_{a}^{\left(k\right)}⊗B_{a}^{\left(k\right)}-(F_{a}^{\left(k\right)}C⊗G_{a}^{\left(k\right)}D)E\right) \end{array}$$

(A.13) $$\begin{array}{l} \frac{\partial F}{\partial \gamma_{k}}=vec{\left(A_{ɛ}\right)}^{T}\left({\tilde{A}}_{a}^{\left(k\right)}⊗{\tilde{B}}_{a}^{\left(k\right)}\right)vec(A_{ɛ})\\ \;\;\;\;\;\;\;\;\;\;\;\;\;\;\;\;\;\;\;\;\;=vec{\left(A_{ɛ}\right)}^{T}vec\left({\tilde{B}}_{a}^{\left(k\right)}A_{ɛ}{\tilde{A}}_{a}^{{\left(k\right)}^{T}}\right)\\ \;\;\;\;\;\;\;\;\;\;\;\;\;\;\;\;\;\;\;\;\;=tr(A_{ɛ}^{T}{\tilde{B}}_{a}^{\left(k\right)}A_{ɛ}{\tilde{A}}_{a}^{{\left(k\right)}^{T}}) \end{array}$$

where we have used *Π*^− 1^ = *Φ*_2_*EΦ*_2_^*T*^ and the notation in Eq. (A.11). The expression in Eq. (A.10) is derived from the expected Fisher information, I_*kn*_ = − 〈∂^2^*F* / ∂*α*_*k*_ ∂*α*_*n*_〉, see Fig. 3 last line, using Eq. (A.12) and the cyclic property of trace. These expressions simplify further using tr(A ⊗ B) = tr(A)tr(B). Note, if the data are transformed, i.e., $\tilde{Y}=P_{r}YP_{c}$, then all variables are transformed as shown in Eq. (6).

The formulation above is not a computationally efficient way to implement the algorithm. We want to make use of $K_{i}=\Phi_{K_{i}}D_{K_{i}}\Phi_{K_{i}}^{T}$ and $S_{i}=\Phi_{S_{i}}D_{S_{i}}\Phi_{S_{i}}^{T}$, in particular, given

(A.14) $$\begin{array}{c} L=\Phi_{K_{2}}\Lambda \Phi_{K_{2}}^{T}\\ \exp(-L\tau )=\Phi_{K_{2}}D_{K_{2}}\Phi_{K_{2}}^{T}\\ D_{K_{2}}=f(\Lambda ,\tau )=\exp(-\Lambda \tau ) \end{array}$$

Computationally efficient expressions are obtained using tr(AB^*T*^) = 1^*T*^(A○B)1 (where ○ is the Hadamard product, 1 is a column of ones) and the following

(A.15) $$\begin{array}{c} \text{tr}({\overset{―}{A}}_{a}^{\left(k\right)})=\text{tr}(A_{a}^{\left(k\right)})\\ \text{tr}({\overset{―}{B}}_{a}^{\left(k\right)})=\text{tr}(B_{a}^{\left(k\right)})\\ {\overset{―}{C}}_{a}^{\left(k\right)}=F_{a}^{\left(k\right)}C\\ {\overset{―}{D}}_{a}^{\left(k\right)}=G_{a}^{\left(k\right)}D\\ {\overset{―}{F}}_{ab}^{\left(kn\right)}=F_{a}^{\left(k\right)}A_{b}^{\left(n\right)}C\\ {\overset{―}{G}}_{ab}^{\left(kn\right)}=G_{a}^{\left(k\right)}B_{b}^{\left(n\right)}D \end{array}$$

together with the expressions in Tables 3−5. Here we have used the notation ${\overset{―}{X}}_{a1b}^{}=\Phi_{a}^{T}X_{1}^{}\Phi_{b}$ to represent left and right multiplication of *X*_1_ by bases *Ф*_*a*_ and *Ф*_*b*_ respectively. This is important when using a reduced eigen system, e.g., *n*_*a*_,*n*_*b*_ < *N*, as the dimension of *X*_1_ is reduced from *N* × *N* to *n*_*a*_ × *n*_*b*_. Components of Eqs. (A.9) and (A.10) then can be written

(A.16) $$\begin{array}{c} tr(A_{a}^{\left(k\right)})=1^{T}({\overset{―}{K}}_{a1a}^{-1}\circ dD_{a}^{\left(k\right)})1\\ tr(B_{a}^{\left(k\right)})=1^{T}({\overset{―}{S}}_{a1a}^{-1}\circ dD_{a}^{\left(k\right)})1\\ tr((F_{a}^{\left(k\right)}C⊗G_{a}^{\left(k\right)}D)E)=1^{T}(({\overset{―}{C}}_{a}^{\left(k\right)}⊗{\overset{―}{D}}_{a}^{\left(k\right)})\circ E^{T})1\\ tr(A_{a}^{\left(k\right)}A_{b}^{\left(n\right)})=1^{T}(({\overset{―}{K}}_{b1a}^{-1}dD_{a}^{\left(k\right)})\circ {\left({\overset{―}{K}}_{a1b}^{-1}dD_{b}^{\left(n\right)}\right)}^{T})1\\ tr(B_{a}^{\left(k\right)}B_{b}^{\left(n\right)})=1^{T}(({\overset{―}{S}}_{b1a}^{-1}dD_{a}^{\left(k\right)})\circ {\left({\overset{―}{S}}_{a1b}^{-1}dD_{b}^{\left(n\right)}\right)}^{T})1\\ tr((F_{a}^{\left(k\right)}C⊗G_{a}^{\left(k\right)}D)E(F_{b}^{\left(n\right)}C⊗G_{b}^{\left(n\right)}D)E)=1^{T}(({\overset{―}{C}}_{a}^{\left(k\right)}⊗{\overset{―}{D}}_{a}^{\left(k\right)})E\circ {\left(({\overset{―}{C}}_{b}^{\left(n\right)}⊗{\overset{―}{D}}_{b}^{\left(n\right)})E\right)}^{T})1\\ tr((F_{a}^{\left(k\right)}A_{b}^{\left(n\right)}C⊗G_{a}^{\left(k\right)}B_{b}^{\left(n\right)}D)E=1^{T}(({\overset{―}{F}}_{ab}^{\left(kn\right)}⊗{\overset{―}{G}}_{ab}^{\left(kn\right)})\circ E^{T})1 \end{array}$$

The expressions for tr(*A*_*a*_^(*k*)^) and tr(*B*_*a*_^(*k*)^) are sparse because *dD*_*a*_^(*k*)^ is diagonal, even if *K*¯_a1a_^–1^ or *S*¯_a1a_^–1^ are not.

## Appendix B Embedding space metric of graph Laplacian

In this paper the embedding metric (Eq. (13)) is fixed, where $H_{d}=I_{n_{d}}$ and

(A.17) $$\begin{array}{l} H_{\text{f}}=C_{ols}^{-1}\\ C_{ols}=\left(\theta_{ols}-M_{ols}\right){\left(\theta_{ols}-M_{ols}\right)}^{T}\\ \mu_{ols}=\text{vec}\left(\theta_{ols}\right)\\ M_{ols}=\frac{1}{N}\theta_{ols}1_{N}1_{N}^{T} \end{array}$$

where *θ*_*ols*_ is the *P *× *N* matrix of OLS estimates and 1_*N*_ is a column vector of ones length *N*.

## Footnotes

1 A univariate random variable (rv), *x*, has probability density function (pdf) (2*πσ*^2^)^−^ ^1/2^exp(− (x − *μ*)^2^ / 2*σ*^2^), *x *∈ R, *μ *∈ R, a multivariate rv, *x *= (*x*_1_,...,*x*_*r*_)^*T*^, has pdf (2*π*)^−^ ^1/2^|*S*|^−^ ^*r*/2^exp(− *tr*(*S*^−^ ^1^(*x* − *μ*)(*x* − *μ*)^*T*^ / 2), *x *∈ R^*r*^^ × 1^, *μ *∈ R^*r*^^ × 1^ and is represented by *x* ~ *N*_*r*_(*μ*,*S*) and a matrix-variate normal rv, *X*, has pdf (2*π*)^−^ ^*rc*/2^|*S*|^−^ ^c / 2^|*K*|^−^ ^*r*^^ / 2^exp(− *tr(S*^−^ ^1^(*X* − *M*)*K*^−^ ^1^(*X* − *M*)^*T*^) / 2), *X *∈ R^*r*^^ × c^, *M* ϵ R^*r*^^ × ^^*c*^ represented by *X *~ *N*_*r,c*_(*M*,*S* ⊗ *K*) with multivariate densities over *X⃗^T^* ~ *N*_rc_(*M⃗^T^*, *S* ⊗ *K*) and *X⃗ ~ N*_cr_(*M⃗*, *S* ⊗ *K*).

2 Technically the Laplacian matrix is a function of the random variable, *b*, i.e., **L**(*b*,*H*), which renders it the generator of a nonlinear dynamic system. This can be approximated by substituting the conditional expectation, *μ* = <*b*>, i.e., **L**(*μ*,*H*), which is used in the main text.

3 This is equivalent to a Newton step, but using the expected curvature as opposed to the local curvature of the objective function.

4 A posterior probability map has two thresholds *t*_1 _ϵ R and *t*_2 _ϵ [0,1] that are used to show voxels where the model is at least 100 × *t*_2_% sure that the effect size is greater than *t*_1_ and is represented by the expression *p*(**β** > *t*_1_) > *t*_2_.

5 The *i*th local kernel is centred at the *i*th voxel and is given by (an image format of) the same row of the spatial covariance matrix.
